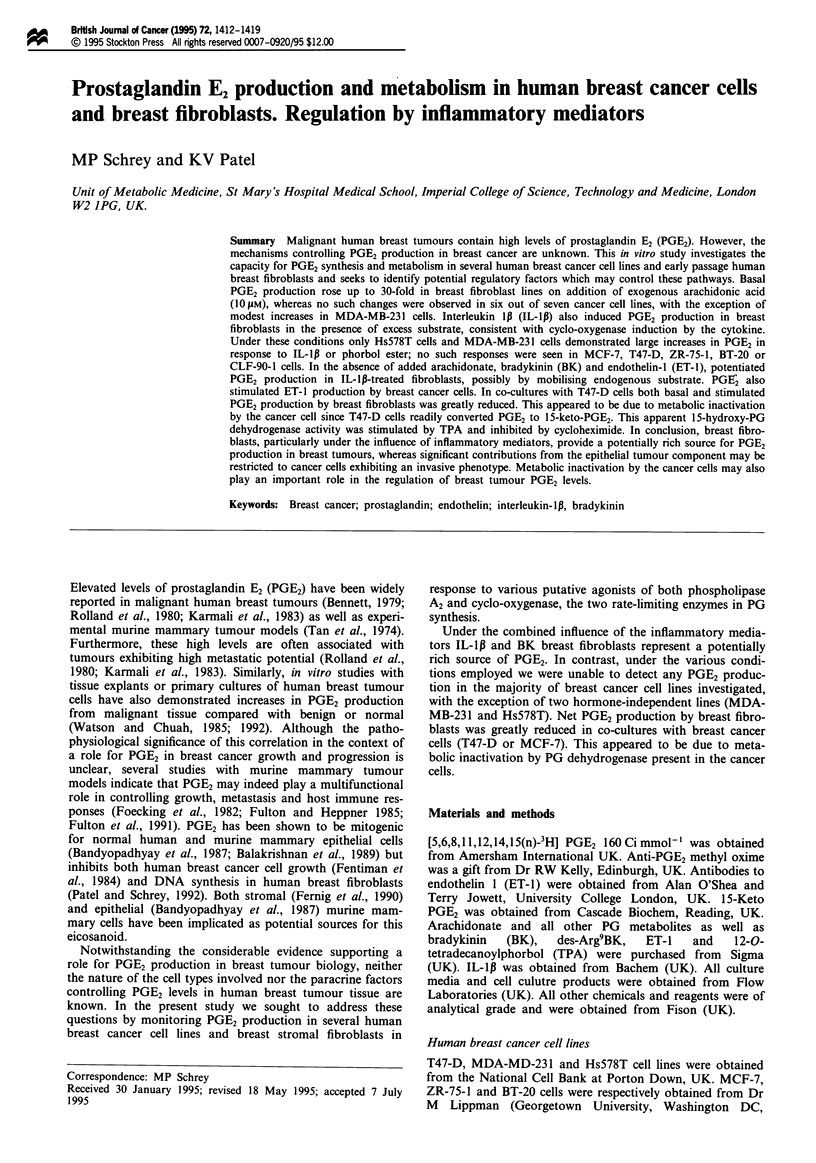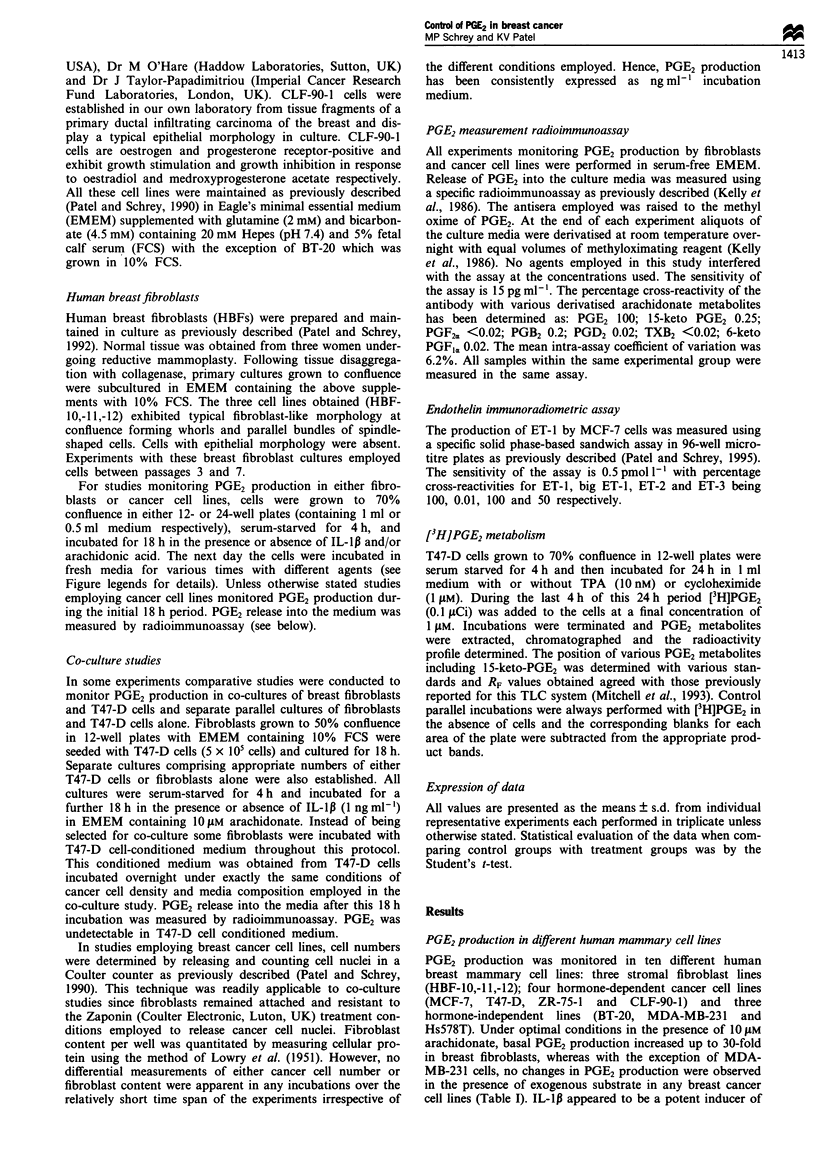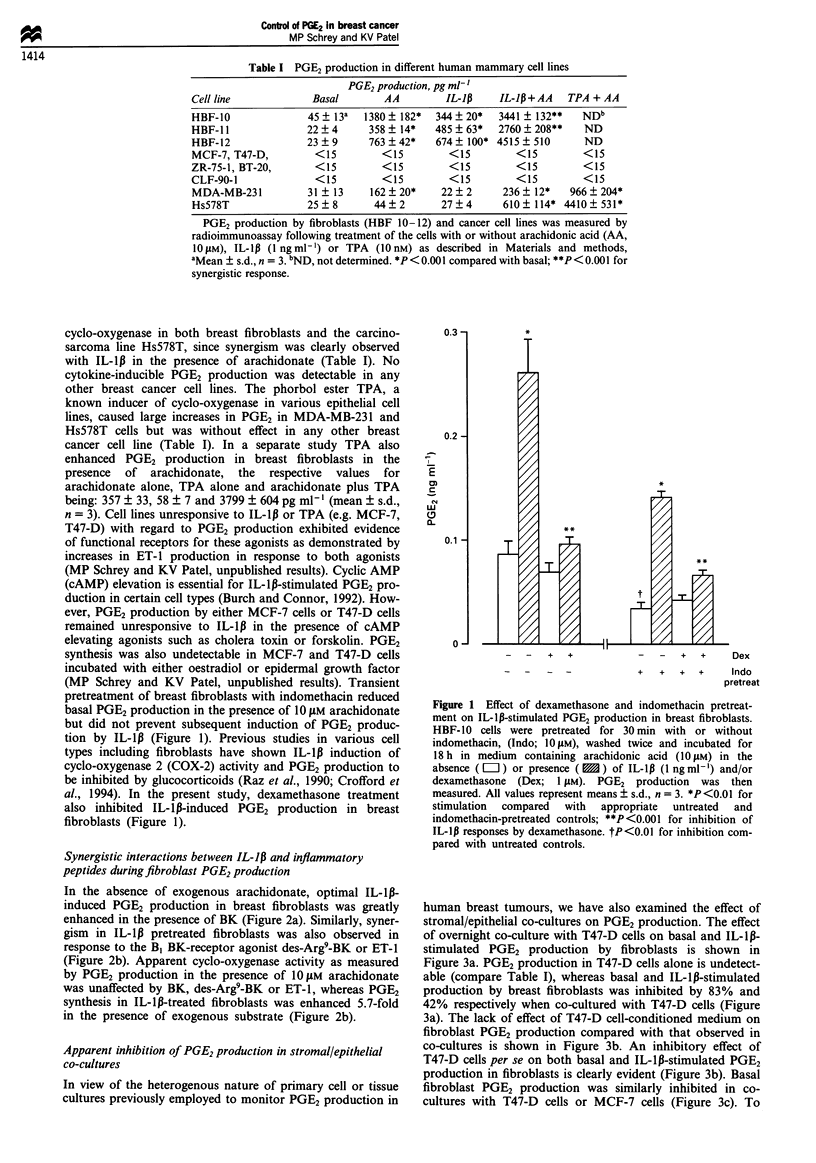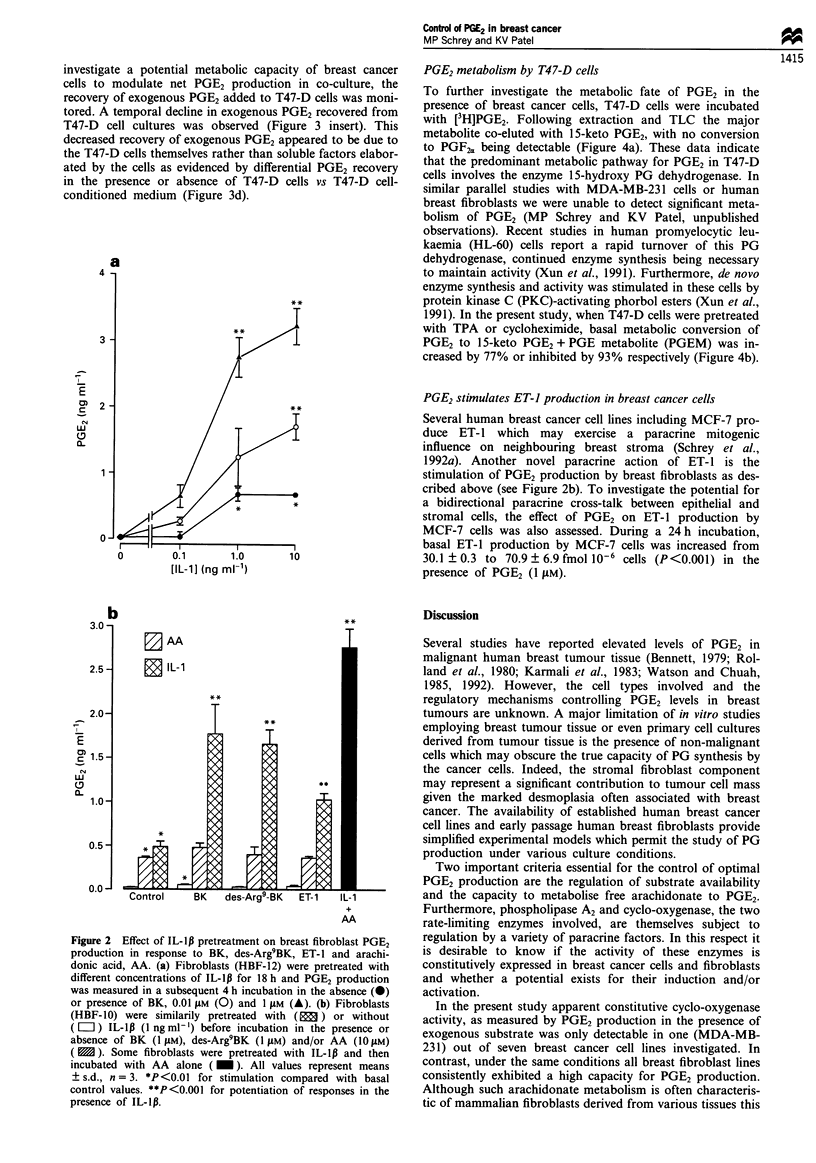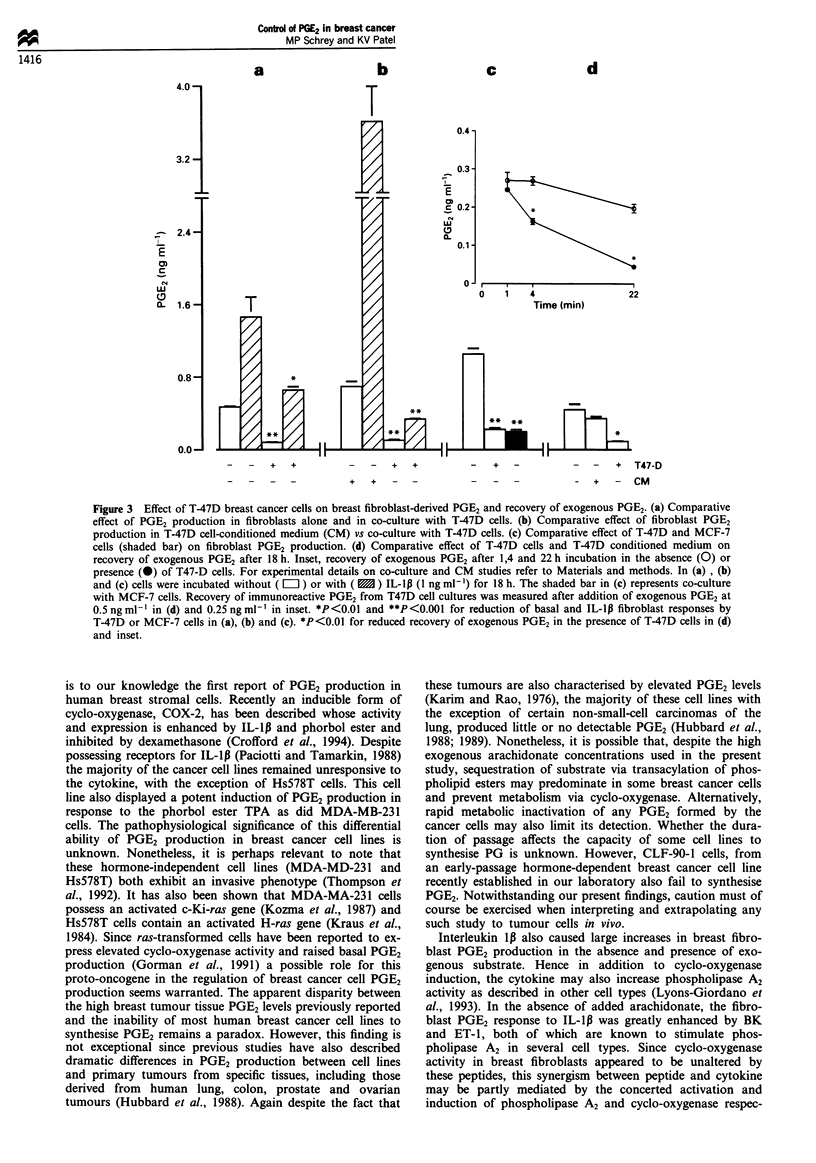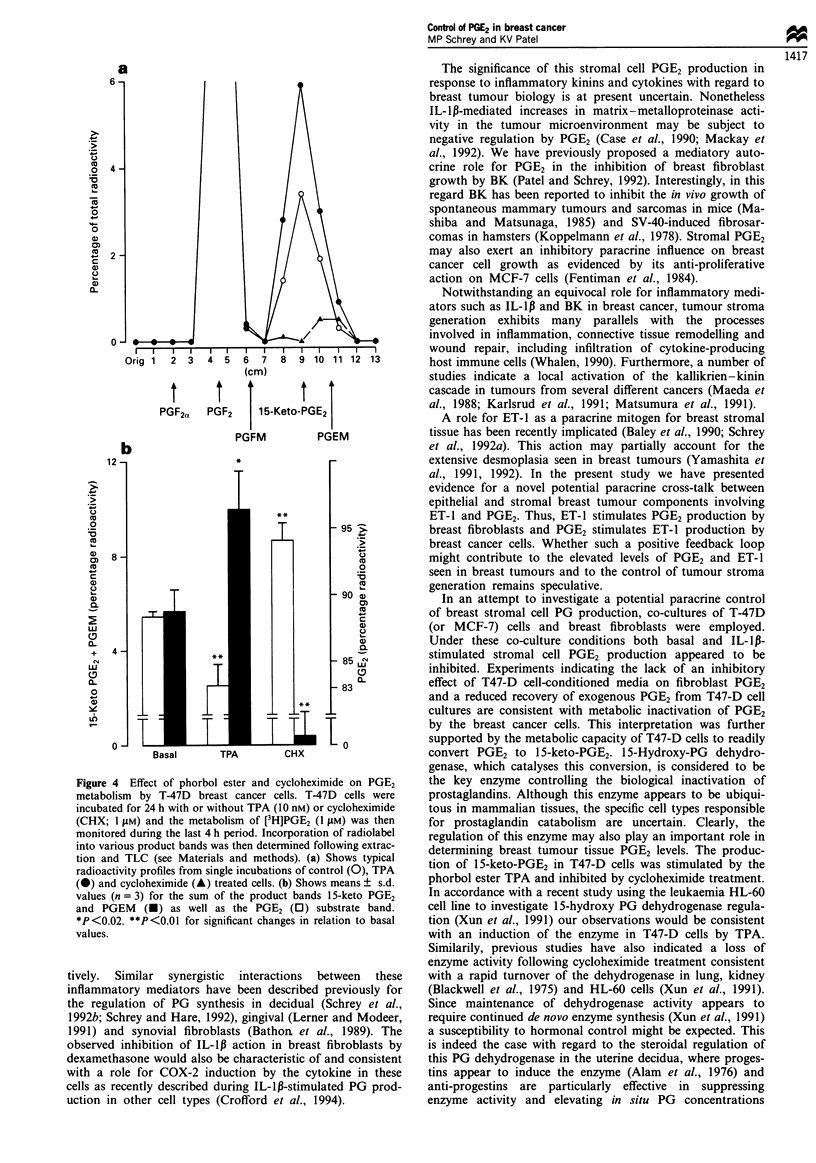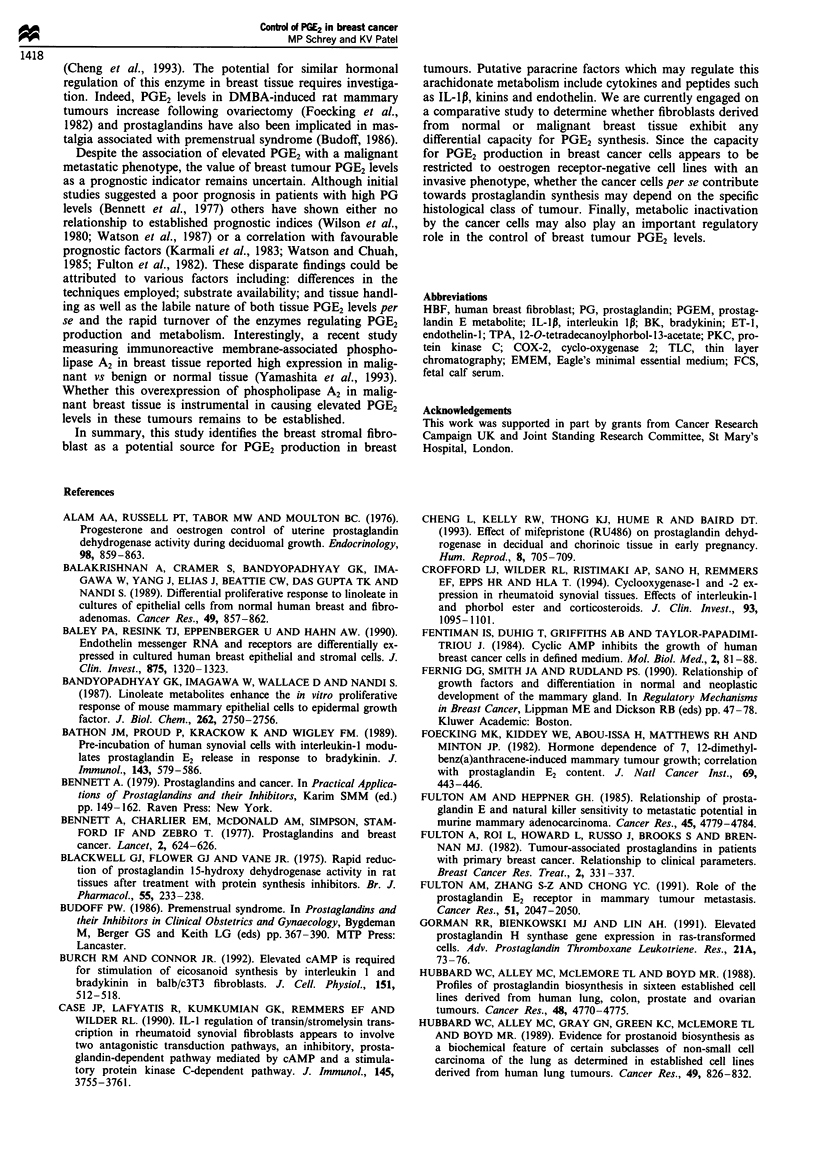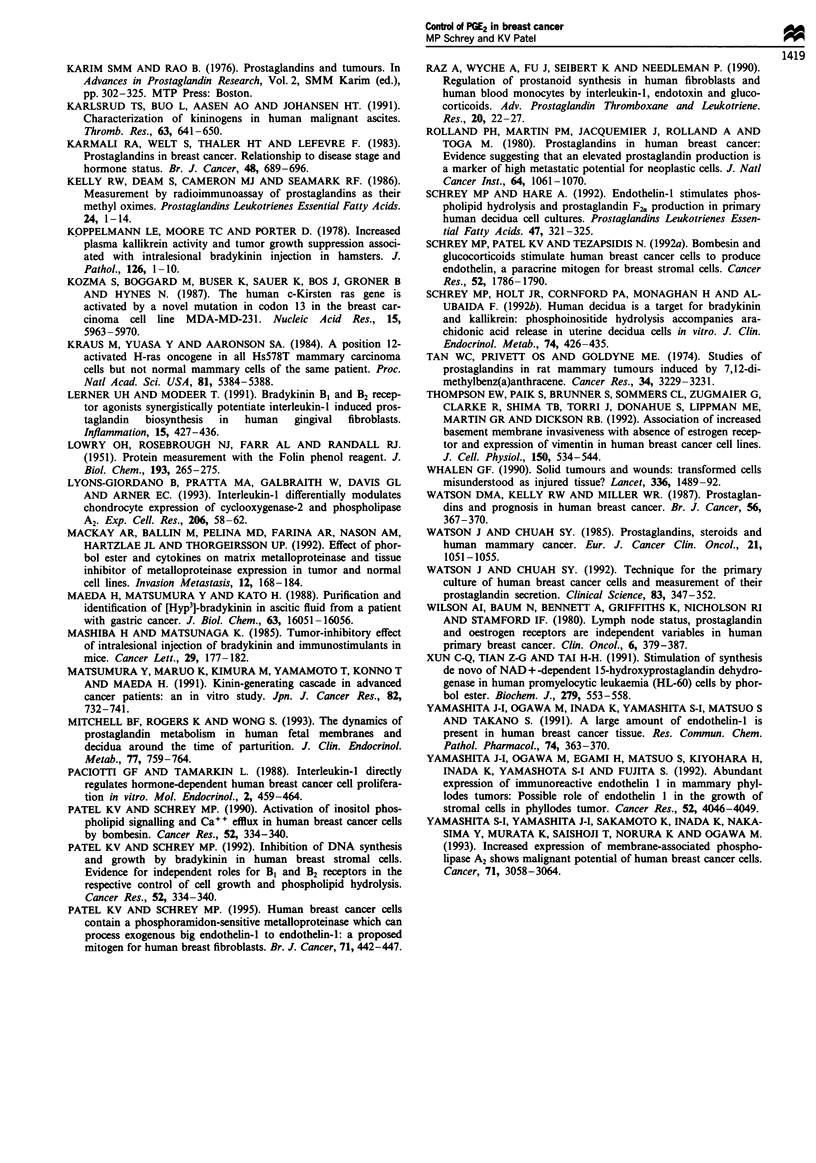# Prostaglandin E2 production and metabolism in human breast cancer cells and breast fibroblasts. Regulation by inflammatory mediators.

**DOI:** 10.1038/bjc.1995.523

**Published:** 1995-12

**Authors:** M. P. Schrey, K. V. Patel

**Affiliations:** Unit of Metabolic Medicine, St Mary's Hospital Medical School, Imperial College of Science, London, UK.

## Abstract

Malignant human breast tumours contain high levels of prostaglandin E2 (PGE2). However, the mechanisms controlling PGE2 production in breast cancer are unknown. This in vitro study investigates the capacity for PGE2 synthesis and metabolism in several human breast cancer cell lines and early passage human breast fibroblasts and seeks to identify potential regulatory factors which may control these pathways. Basal PGE2 production rose up to 30-fold in breast fibroblast lines on addition of exogenous arachidonic acid (10 microM), whereas no such changes were observed in six out of seven cancer cell lines, with the exception of modest increases in MDA-MB-231 cells. Interleukin 1 beta (IL-1 beta) also induced PGE2 production in breast fibroblasts in the presence of excess substrate, consistent with cyclo-oxygenase induction by the cytokine. Under these conditions only Hs578T cells and MDA-MB-231 cells demonstrated large increases in PGE2 in response to IL-1 beta or phorbol ester; no such responses were seen in MCF-7, T47-D, ZR-75-1, BT-20 or CLF-90-1 cells. In the absence of added arachidonate, bradykinin (BK) and endothelin-1 (ET-1), potentiated PGE2 production in IL-1 beta-treated fibroblasts, possibly by mobilising endogenous substrate. PGE2 also stimulated ET-1 production by breast cancer cells. In co-cultures with T47-D cells both basal and stimulated PGE2 production by breast fibroblasts was greatly reduced. This appeared to be due to metabolic inactivation by the cancer cell since T47-D cells readily converted PGE2 to 15-keto-PGE2. This apparent 15-hydroxy-PG dehydrogenase activity was stimulated by TPA and inhibited by cycloheximide. In conclusion, breast fibroblasts, particularly under the influence of inflammatory mediators, provide a potentially rich source for PGE2 production in breast tumours, whereas significant contributions from the epithelial tumour component may be restricted to cancer cells exhibiting an invasive phenotype. Metabolic inactivation by the cancer cells may also play an important role in the regulation of breast tumour PGE2 levels.


British Journal of Cancer (1995) 72, 1412-1419
© 1995 Stockton Press All rights reserved 0007-0920/95 $12.00

### Prostaglandin $E_2$ production and metabolism in human breast cancer cells and breast fibroblasts. Regulation by inflammatory mediators

MP Schrey and KV Patel

*Unit of Metabolic Medicine, St Mary's Hospital Medical School, Imperial College of Science, Technology and Medicine, London W2 1PG, UK.*

**Summary** Malignant human breast tumours contain high levels of prostaglandin $E_2$ ($PGE_2$). However, the mechanisms controlling $PGE_2$ production in breast cancer are unknown. This *in vitro* study investigates the capacity for $PGE_2$ synthesis and metabolism in several human breast cancer cell lines and early passage human breast fibroblasts and seeks to identify potential regulatory factors which may control these pathways. Basal $PGE_2$ production rose up to 30-fold in breast fibroblast lines on addition of exogenous arachidonic acid (10 μM), whereas no such changes were observed in six out of seven cancer cell lines, with the exception of modest increases in MDA-MB-231 cells. Interleukin 1β (IL-1β) also induced $PGE_2$ production in breast fibroblasts in the presence of excess substrate, consistent with cyclo-oxygenase induction by the cytokine. Under these conditions only Hs578T cells and MDA-MB-231 cells demonstrated large increases in $PGE_2$ in response to IL-1β or phorbol ester; no such responses were seen in MCF-7, T47-D, ZR-75-1, BT-20 or CLF-90-1 cells. In the absence of added arachidonate, bradykinin (BK) and endothelin-1 (ET-1), potentiated $PGE_2$ production in IL-1β-treated fibroblasts, possibly by mobilising endogenous substrate. $PGE_2$ also stimulated ET-1 production by breast cancer cells. In co-cultures with T47-D cells both basal and stimulated $PGE_2$ production by breast fibroblasts was greatly reduced. This appeared to be due to metabolic inactivation by the cancer cell since T47-D cells readily converted $PGE_2$ to 15-keto-$PGE_2$. This apparent 15-hydroxy-PG dehydrogenase activity was stimulated by TPA and inhibited by cycloheximide. In conclusion, breast fibroblasts, particularly under the influence of inflammatory mediators, provide a potentially rich source for $PGE_2$ production in breast tumours, whereas significant contributions from the epithelial tumour component may be restricted to cancer cells exhibiting an invasive phenotype. Metabolic inactivation by the cancer cells may also play an important role in the regulation of breast tumour $PGE_2$ levels.

**Keywords:** Breast cancer; prostaglandin; endothelin; interleukin-1β, bradykinin

Elevated levels of prostaglandin $E_2$ ($PGE_2$) have been widely reported in malignant human breast tumours (Bennett, 1979; Rolland *et al.*, 1980; Karmali *et al.*, 1983) as well as experimental murine mammary tumour models (Tan *et al.*, 1974). Furthermore, these high levels are often associated with tumours exhibiting high metastatic potential (Rolland *et al.*, 1980; Karmali *et al.*, 1983). Similarly, *in vitro* studies with tissue explants or primary cultures of human breast tumour cells have also demonstrated increases in $PGE_2$ production from malignant tissue compared with benign or normal (Watson and Chuah, 1985; 1992). Although the pathophysiological significance of this correlation in the context of a role for $PGE_2$ in breast cancer growth and progression is unclear, several studies with murine mammary tumour models indicate that $PGE_2$ may indeed play a multifunctional role in controlling growth, metastasis and host immune responses (Foecking *et al.*, 1982; Fulton and Heppner 1985; Fulton *et al.*, 1991). $PGE_2$ has been shown to be mitogenic for normal human and murine mammary epithelial cells (Bandyopadhyay *et al.*, 1987; Balakrishnan *et al.*, 1989) but inhibits both human breast cancer cell growth (Fentiman *et al.*, 1984) and DNA synthesis in human breast fibroblasts (Patel and Schrey, 1992). Both stromal (Fernig *et al.*, 1990) and epithelial (Bandyopadhyay *et al.*, 1987) murine mammary cells have been implicated as potential sources for this eicosanoid.

Notwithstanding the considerable evidence supporting a role for $PGE_2$ production in breast tumour biology, neither the nature of the cell types involved nor the paracrine factors controlling $PGE_2$ levels in human breast tumour tissue are known. In the present study we sought to address these questions by monitoring $PGE_2$ production in several human breast cancer cell lines and breast stromal fibroblasts in response to various putative agonists of both phospholipase $A_2$ and cyclo-oxygenase, the two rate-limiting enzymes in PG synthesis.

Under the combined influence of the inflammatory mediators IL-1β and BK breast fibroblasts represent a potentially rich source of $PGE_2$. In contrast, under the various conditions employed we were unable to detect any $PGE_2$ production in the majority of breast cancer cell lines investigated, with the exception of two hormone-independent lines (MDA-MB-231 and Hs578T). Net $PGE_2$ production by breast fibroblasts was greatly reduced in co-cultures with breast cancer cells (T47-D or MCF-7). This appeared to be due to metabolic inactivation by PG dehydrogenase present in the cancer cells.

### Materials and methods

[5,6,8,11,12,14,15(n)-$^3$H] $PGE_2$ 160 Ci $mmol^{-1}$ was obtained from Amersham International UK. Anti-$PGE_2$ methyl oxime was a gift from Dr RW Kelly, Edinburgh, UK. Antibodies to endothelin 1 (ET-1) were obtained from Alan O'Shea and Terry Jowett, University College London, UK. 15-Keto $PGE_2$ was obtained from Cascade Biochem, Reading, UK. Arachidonate and all other PG metabolites as well as bradykinin (BK), des-Arg$^9$BK, ET-1 and 12-*O*-tetradecanoylphorbol (TPA) were purchased from Sigma (UK). IL-1β was obtained from Bachem (UK). All culture media and cell culutre products were obtained from Flow Laboratories (UK). All other chemicals and reagents were of analytical grade and were obtained from Fison (UK).

### *Human breast cancer cell lines*

T47-D, MDA-MD-231 and Hs578T cell lines were obtained from the National Cell Bank at Porton Down, UK. MCF-7, ZR-75-1 and BT-20 cells were respectively obtained from Dr M Lippman (Georgetown University, Washington DC,

Correspondence: MP Schrey
Received 30 January 1995; revised 18 May 1995; accepted 7 July 1995

USA), Dr M O'Hare (Haddow Laboratories, Sutton, UK) and Dr J Taylor-Papadimitriou (Imperial Cancer Research Fund Laboratories, London, UK). CLF-90-1 cells were established in our own laboratory from tissue fragments of a primary ductal infiltrating carcinoma of the breast and display a typical epithelial morphology in culture. CLF-90-1 cells are oestrogen and progesterone receptor-positive and exhibit growth stimulation and growth inhibition in response to oestradiol and medroxyprogesterone acetate respectively. All these cell lines were maintained as previously described (Patel and Schrey, 1990) in Eagle's minimal essential medium (EMEM) supplemented with glutamine (2 mM) and bicarbonate (4.5 mM) containing 20 mM Hepes (pH 7.4) and 5% fetal calf serum (FCS) with the exception of BT-20 which was grown in 10% FCS.

### *Human breast fibroblasts*

Human breast fibroblasts (HBFs) were prepared and maintained in culture as previously described (Patel and Schrey, 1992). Normal tissue was obtained from three women undergoing reductive mammoplasty. Following tissue disaggregation with collagenase, primary cultures grown to confluence were subcultured in EMEM containing the above supplements with 10% FCS. The three cell lines obtained (HBF-10,-11,-12) exhibited typical fibroblast-like morphology at confluence forming whorls and parallel bundles of spindle-shaped cells. Cells with epithelial morphology were absent. Experiments with these breast fibroblast cultures employed cells between passages 3 and 7.

For studies monitoring PGE$_2$ production in either fibroblasts or cancer cell lines, cells were grown to 70% confluence in either 12- or 24-well plates (containing 1 ml or 0.5 ml medium respectively), serum-starved for 4 h, and incubated for 18 h in the presence or absence of IL-1$\beta$ and/or arachidonic acid. The next day the cells were incubated in fresh media for various times with different agents (see Figure legends for details). Unless otherwise stated studies employing cancer cell lines monitored PGE$_2$ production during the initial 18 h period. PGE$_2$ release into the medium was measured by radioimmunoassay (see below).

### *Co-culture studies*

In some experiments comparative studies were conducted to monitor PGE$_2$ production in co-cultures of breast fibroblasts and T47-D cells and separate parallel cultures of fibroblasts and T47-D cells alone. Fibroblasts grown to 50% confluence in 12-well plates with EMEM containing 10% FCS were seeded with T47-D cells ($5 \times 10^5$ cells) and cultured for 18 h. Separate cultures comprising appropriate numbers of either T47-D cells or fibroblasts alone were also established. All cultures were serum-starved for 4 h and incubated for a further 18 h in the presence or absence of IL-1$\beta$ (1 ng ml$^{-1}$) in EMEM containing 10 $\mu$M arachidonate. Instead of being selected for co-culture some fibroblasts were incubated with T47-D cell-conditioned medium throughout this protocol. This conditioned medium was obtained from T47-D cells incubated overnight under exactly the same conditions of cancer cell density and media composition employed in the co-culture study. PGE$_2$ release into the media after this 18 h incubation was measured by radioimmunoassay. PGE$_2$ was undetectable in T47-D cell conditioned medium.

In studies employing breast cancer cell lines, cell numbers were determined by releasing and counting cell nuclei in a Coulter counter as previously described (Patel and Schrey, 1990). This technique was readily applicable to co-culture studies since fibroblasts remained attached and resistant to the Zaponin (Coulter Electronic, Luton, UK) treatment conditions employed to release cancer cell nuclei. Fibroblast content per well was quantitated by measuring cellular protein using the method of Lowry *et al.* (1951). However, no differential measurements of either cancer cell number or fibroblast content were apparent in any incubations over the relatively short time span of the experiments irrespective of the different conditions employed. Hence, PGE$_2$ production has been consistently expressed as ng ml$^{-1}$ incubation medium.

### *PGE$_2$ measurement radioimmunoassay*

All experiments monitoring PGE$_2$ production by fibroblasts and cancer cell lines were performed in serum-free EMEM. Release of PGE$_2$ into the culture media was measured using a specific radioimmunoassay as previously described (Kelly *et al.*, 1986). The antisera employed was raised to the methyl oxime of PGE$_2$. At the end of each experiment aliquots of the culture media were derivatised at room temperature overnight with equal volumes of methyloximating reagent (Kelly *et al.*, 1986). No agents employed in this study interfered with the assay at the concentrations used. The sensitivity of the assay is 15 pg ml$^{-1}$. The percentage cross-reactivity of the antibody with various derivatised arachidonate metabolites has been determined as: PGE$_2$ 100; 15-keto PGE$_2$ 0.25; PGF$_{2\alpha}$ <0.02; PGB$_2$ 0.2; PGD$_2$ 0.02; TXB$_2$ <0.02; 6-keto PGF$_{1\alpha}$ 0.02. The mean intra-assay coefficient of variation was 6.2%. All samples within the same experimental group were measured in the same assay.

### *Endothelin immunoradiometric assay*

The production of ET-1 by MCF-7 cells was measured using a specific solid phase-based sandwich assay in 96-well microtitre plates as previously described (Patel and Schrey, 1995). The sensitivity of the assay is 0.5 pmol l$^{-1}$ with percentage cross-reactivities for ET-1, big ET-1, ET-2 and ET-3 being 100, 0.01, 100 and 50 respectively.

### *[$^3$H]PGE$_2$ metabolism*

T47-D cells grown to 70% confluence in 12-well plates were serum starved for 4 h and then incubated for 24 h in 1 ml medium with or without TPA (10 nM) or cycloheximide (1 $\mu$M). During the last 4 h of this 24 h period [$^3$H]PGE$_2$ (0.1 $\mu$Ci) was added to the cells at a final concentration of 1 $\mu$M. Incubations were terminated and PGE$_2$ metabolites were extracted, chromatographed and the radioactivity profile determined. The position of various PGE$_2$ metabolites including 15-keto-PGE$_2$ was determined with various standards and $R_F$ values obtained agreed with those previously reported for this TLC system (Mitchell *et al.*, 1993). Control parallel incubations were always performed with [$^3$H]PGE$_2$ in the absence of cells and the corresponding blanks for each area of the plate were subtracted from the appropriate product bands.

### *Expression of data*

All values are presented as the means ± s.d. from individual representative experiments each performed in triplicate unless otherwise stated. Statistical evaluation of the data when comparing control groups with treatment groups was by the Student's *t*-test.

### Results

### *PGE$_2$ production in different human mammary cell lines*

PGE$_2$ production was monitored in ten different human breast mammary cell lines: three stromal fibroblast lines (HBF-10,-11,-12); four hormone-dependent cancer cell lines (MCF-7, T47-D, ZR-75-1 and CLF-90-1) and three hormone-independent lines (BT-20, MDA-MB-231 and Hs578T). Under optimal conditions in the presence of 10 $\mu$M arachidonate, basal PGE$_2$ production increased up to 30-fold in breast fibroblasts, whereas with the exception of MDA-MB-231 cells, no changes in PGE$_2$ production were observed in the presence of exogenous substrate in any breast cancer cell lines (Table I). IL-1$\beta$ appeared to be a potent inducer of

**Table I** $PGE_2$ production in different human mammary cell lines

| Cell line | PGE₂ production, pg ml⁻¹ Basal | AA | IL-1β | IL-1β+AA | TPA+AA |
|---|---|---|---|---|---|
| HBF-10 | 45 ± 13[a] | 1380 ± 182* | 344 ± 20* | 3441 ± 132** | ND[b] |
| HBF-11 | 22 ± 4 | 358 ± 14* | 485 ± 63* | 2760 ± 208** | ND |
| HBF-12 | 23 ± 9 | 763 ± 42* | 674 ± 100* | 4515 ± 510 | ND |
| MCF-7, T47-D, | <15 | <15 | <15 | <15 | <15 |
| ZR-75-1, BT-20, | <15 | <15 | <15 | <15 | <15 |
| CLF-90-1 | <15 | <15 | <15 | <15 | <15 |
| MDA-MB-231 | 31 ± 13 | 162 ± 20* | 22 ± 2 | 236 ± 12* | 966 ± 204* |
| Hs578T | 25 ± 8 | 44 ± 2 | 27 ± 4 | 610 ± 114* | 4410 ± 531* |

$PGE_2$ production by fibroblasts (HBF 10–12) and cancer cell lines was measured by radioimmunoassay following treatment of the cells with or without arachidonic acid (AA, 10 μM), IL-1β (1 ng ml$^{-1}$) or TPA (10 nM) as described in Materials and methods, [a]Mean ± s.d., $n = 3$. [b]ND, not determined. *$P < 0.001$ compared with basal; **$P < 0.001$ for synergistic response.

cyclo-oxygenase in both breast fibroblasts and the carcinosarcoma line Hs578T, since synergism was clearly observed with IL-1β in the presence of arachidonate (Table I). No cytokine-inducible $PGE_2$ production was detectable in any other breast cancer cell lines. The phorbol ester TPA, a known inducer of cyclo-oxygenase in various epithelial cell lines, caused large increases in $PGE_2$ in MDA-MB-231 and Hs578T cells but was without effect in any other breast cancer cell line (Table I). In a separate study TPA also enhanced $PGE_2$ production in breast fibroblasts in the presence of arachidonate, the respective values for arachidonate alone, TPA alone and arachidonate plus TPA being: 357 ± 33, 58 ± 7 and 3799 ± 604 pg ml$^{-1}$ (mean ± s.d., $n = 3$). Cell lines unresponsive to IL-1β or TPA (e.g. MCF-7, T47-D) with regard to $PGE_2$ production exhibited evidence of functional receptors for these agonists as demonstrated by increases in ET-1 production in response to both agonists (MP Schrey and KV Patel, unpublished results). Cyclic AMP (cAMP) elevation is essential for IL-1β-stimulated $PGE_2$ production in certain cell types (Burch and Connor, 1992). However, $PGE_2$ production by either MCF-7 cells or T47-D cells remained unresponsive to IL-1β in the presence of cAMP elevating agonists such as cholera toxin or forskolin. $PGE_2$ synthesis was also undetectable in MCF-7 and T47-D cells incubated with either oestradiol or epidermal growth factor (MP Schrey and KV Patel, unpublished results). Transient pretreatment of breast fibroblasts with indomethacin reduced basal $PGE_2$ production in the presence of 10 μM arachidonate but did not prevent subsequent induction of $PGE_2$ production by IL-1β (Figure 1). Previous studies in various cell types including fibroblasts have shown IL-1β induction of cyclo-oxygenase 2 (COX-2) activity and $PGE_2$ production to be inhibited by glucocorticoids (Raz *et al*., 1990; Crofford *et al*., 1994). In the present study, dexamethasone treatment also inhibited IL-1β-induced $PGE_2$ production in breast fibroblasts (Figure 1).

**Figure 1** Effect of dexamethasone and indomethacin pretreatment on IL-1β-stimulated $PGE_2$ production in breast fibroblasts. HBF-10 cells were pretreated for 30 min with or without indomethacin, (Indo; 10 μM), washed twice and incubated for 18 h in medium containing arachidonic acid (10 μM) in the absence (□) or presence (▨) of IL-1β (1 ng ml$^{-1}$) and/or dexamethasone (Dex; 1 μM). $PGE_2$ production was then measured. All values represent means ± s.d., $n = 3$. *$P<0.01$ for stimulation compared with appropriate untreated and indomethacin-pretreated controls; **$P<0.001$ for inhibition of IL-1β responses by dexamethasone. †$P<0.01$ for inhibition compared with untreated controls.

*Synergistic interactions between IL-1β and inflammatory peptides during fibroblast $PGE_2$ production*

In the absence of exogenous arachidonate, optimal IL-1β-induced $PGE_2$ production in breast fibroblasts was greatly enhanced in the presence of BK (Figure 2a). Similarly, synergism in IL-1β pretreated fibroblasts was also observed in response to the $B_1$ BK-receptor agonist des-Arg$^9$-BK or ET-1 (Figure 2b). Apparent cyclo-oxygenase activity as measured by $PGE_2$ production in the presence of 10 μM arachidonate was unaffected by BK, des-Arg$^9$-BK or ET-1, whereas $PGE_2$ synthesis in IL-1β-treated fibroblasts was enhanced 5.7-fold in the presence of exogenous substrate (Figure 2b).

*Apparent inhibition of $PGE_2$ production in stromal/epithelial co-cultures*

In view of the heterogenous nature of primary cell or tissue cultures previously employed to monitor $PGE_2$ production in human breast tumours, we have also examined the effect of stromal/epithelial co-cultures on $PGE_2$ production. The effect of overnight co-culture with T47-D cells on basal and IL-1β-stimulated $PGE_2$ production by fibroblasts is shown in Figure 3a. $PGE_2$ production in T47-D cells alone is undetectable (compare Table I), whereas basal and IL-1β-stimulated production by breast fibroblasts was inhibited by 83% and 42% respectively when co-cultured with T47-D cells (Figure 3a). The lack of effect of T47-D cell-conditioned medium on fibroblast $PGE_2$ production compared with that observed in co-cultures is shown in Figure 3b. An inhibitory effect of T47-D cells *per se* on both basal and IL-1β-stimulated $PGE_2$ production in fibroblasts is clearly evident (Figure 3b). Basal fibroblast $PGE_2$ production was similarly inhibited in co-cultures with T47-D cells or MCF-7 cells (Figure 3c). To investigate a potential metabolic capacity of breast cancer cells to modulate net $PGE_2$ production in co-culture, the recovery of exogenous $PGE_2$ added to T47-D cells was monitored. A temporal decline in exogenous $PGE_2$ recovered from T47-D cell cultures was observed (Figure 3 insert). This decreased recovery of exogenous $PGE_2$ appeared to be due to the T47-D cells themselves rather than soluble factors elaborated by the cells as evidenced by differential $PGE_2$ recovery in the presence or absence of T47-D cells *vs* T47-D cell-conditioned medium (Figure 3d).

**Figure 2** Effect of IL-1$\beta$ pretreatment on breast fibroblast $PGE_2$ production in response to BK, des-Arg$^9$BK, ET-1 and arachidonic acid, AA. (**a**) Fibroblasts (HBF-12) were pretreated with different concentrations of IL-1$\beta$ for 18 h and $PGE_2$ production was measured in a subsequent 4 h incubation in the absence (●) or presence of BK, 0.01 $\mu$M (○) and 1 $\mu$M (▲). (**b**) Fibroblasts (HBF-10) were similarily pretreated with (▩) or without (□) IL-1$\beta$ (1 ng ml$^{-1}$) before incubation in the presence or absence of BK (1 $\mu$M), des-Arg$^9$BK (1 $\mu$M) and/or AA (10 $\mu$M) (▨). Some fibroblasts were pretreated with IL-1$\beta$ and then incubated with AA alone (■). All values represent means ± s.d., $n = 3$. *$P<0.01$ for stimulation compared with basal control values. **$P<0.001$ for potentiation of responses in the presence of IL-1$\beta$.

*$PGE_2$ metabolism by T47-D cells*

To further investigate the metabolic fate of $PGE_2$ in the presence of breast cancer cells, T47-D cells were incubated with [$^3$H]$PGE_2$. Following extraction and TLC the major metabolite co-eluted with 15-keto $PGE_2$, with no conversion to $PGF_{2\alpha}$ being detectable (Figure 4a). These data indicate that the predominant metabolic pathway for $PGE_2$ in T47-D cells involves the enzyme 15-hydroxy PG dehydrogenase. In similar parallel studies with MDA-MB-231 cells or human breast fibroblasts we were unable to detect significant metabolism of $PGE_2$ (MP Schrey and KV Patel, unpublished observations). Recent studies in human promyelocytic leukaemia (HL-60) cells report a rapid turnover of this PG dehydrogenase, continued enzyme synthesis being necessary to maintain activity (Xun *et al.*, 1991). Furthermore, *de novo* enzyme synthesis and activity was stimulated in these cells by protein kinase C (PKC)-activating phorbol esters (Xun *et al.*, 1991). In the present study, when T47-D cells were pretreated with TPA or cycloheximide, basal metabolic conversion of $PGE_2$ to 15-keto $PGE_2$ + PGE metabolite (PGEM) was increased by 77% or inhibited by 93% respectively (Figure 4b).

*$PGE_2$ stimulates ET-1 production in breast cancer cells*

Several human breast cancer cell lines including MCF-7 produce ET-1 which may exercise a paracrine mitogenic influence on neighbouring breast stroma (Schrey *et al.*, 1992*a*). Another novel paracrine action of ET-1 is the stimulation of $PGE_2$ production by breast fibroblasts as described above (see Figure 2b). To investigate the potential for a bidirectional paracrine cross-talk between epithelial and stromal cells, the effect of $PGE_2$ on ET-1 production by MCF-7 cells was also assessed. During a 24 h incubation, basal ET-1 production by MCF-7 cells was increased from $30.1 \pm 0.3$ to $70.9 \pm 6.9$ fmol $10^{-6}$ cells ($P<0.001$) in the presence of $PGE_2$ (1 $\mu$M).

### Discussion

Several studies have reported elevated levels of $PGE_2$ in malignant human breast tumour tissue (Bennett, 1979; Rolland *et al.*, 1980; Karmali *et al.*, 1983; Watson and Chuah, 1985, 1992). However, the cell types involved and the regulatory mechanisms controlling $PGE_2$ levels in breast tumours are unknown. A major limitation of *in vitro* studies employing breast tumour tissue or even primary cell cultures derived from tumour tissue is the presence of non-malignant cells which may obscure the true capacity of PG synthesis by the cancer cells. Indeed, the stromal fibroblast component may represent a significant contribution to tumour cell mass given the marked desmoplasia often associated with breast cancer. The availability of established human breast cancer cell lines and early passage human breast fibroblasts provide simplified experimental models which permit the study of PG production under various culture conditions.

Two important criteria essential for the control of optimal $PGE_2$ production are the regulation of substrate availability and the capacity to metabolise free arachidonate to $PGE_2$. Furthermore, phospholipase $A_2$ and cyclo-oxygenase, the two rate-limiting enzymes involved, are themselves subject to regulation by a variety of paracrine factors. In this respect it is desirable to know if the activity of these enzymes is constitutively expressed in breast cancer cells and fibroblasts and whether a potential exists for their induction and/or activation.

In the present study apparent constitutive cyclo-oxygenase activity, as measured by $PGE_2$ production in the presence of exogenous substrate was only detectable in one (MDA-MB-231) out of seven breast cancer cell lines investigated. In contrast, under the same conditions all breast fibroblast lines consistently exhibited a high capacity for $PGE_2$ production. Although such arachidonate metabolism is often characteristic of mammalian fibroblasts derived from various tissues this

**Figure 3** Effect of T-47D breast cancer cells on breast fibroblast-derived $PGE_2$ and recovery of exogenous $PGE_2$. (**a**) Comparative effect of $PGE_2$ production in fibroblasts alone and in co-culture with T-47D cells. (**b**) Comparative effect of fibroblast $PGE_2$ production in T-47D cell-conditioned medium (CM) *vs* co-culture with T-47D cells. (**c**) Comparative effect of T-47D and MCF-7 cells (shaded bar) on fibroblast $PGE_2$ production. (**d**) Comparative effect of T-47D cells and T-47D conditioned medium on recovery of exogenous $PGE_2$ after 18 h. Inset, recovery of exogenous $PGE_2$ after 1,4 and 22 h incubation in the absence (○) or presence (●) of T47-D cells. For experimental details on co-culture and CM studies refer to Materials and methods. In (**a**), (**b**) and (**c**) cells were incubated without (□) or with (▨) IL-1$\beta$ (1 ng ml$^{-1}$) for 18 h. The shaded bar in (c) represents co-culture with MCF-7 cells. Recovery of immunoreactive $PGE_2$ from T47D cell cultures was measured after addition of exogenous $PGE_2$ at 0.5 ng ml$^{-1}$ in (**d**) and 0.25 ng ml$^{-1}$ in inset. $^*P<0.01$ and $^{**}P<0.001$ for reduction of basal and IL-1$\beta$ fibroblast responses by T-47D or MCF-7 cells in (**a**), (**b**) and (**c**). $^*P<0.01$ for reduced recovery of exogenous $PGE_2$ in the presence of T-47D cells in (**d**) and inset.

is to our knowledge the first report of $PGE_2$ production in human breast stromal cells. Recently an inducible form of cyclo-oxygenase, COX-2, has been described whose activity and expression is enhanced by IL-1$\beta$ and phorbol ester and inhibited by dexamethasone (Crofford *et al.*, 1994). Despite possessing receptors for IL-1$\beta$ (Paciotti and Tamarkin, 1988) the majority of the cancer cell lines remained unresponsive to the cytokine, with the exception of Hs578T cells. This cell line also displayed a potent induction of $PGE_2$ production in response to the phorbol ester TPA as did MDA-MB-231 cells. The pathophysiological significance of this differential ability of $PGE_2$ production in breast cancer cell lines is unknown. Nonetheless, it is perhaps relevant to note that these hormone-independent cell lines (MDA-MD-231 and Hs578T) both exhibit an invasive phenotype (Thompson *et al.*, 1992). It has also been shown that MDA-MA-231 cells possess an activated c-Ki-*ras* gene (Kozma *et al.*, 1987) and Hs578T cells contain an activated H-*ras* gene (Kraus *et al.*, 1984). Since *ras*-transformed cells have been reported to express elevated cyclo-oxygenase activity and raised basal $PGE_2$ production (Gorman *et al.*, 1991) a possible role for this proto-oncogene in the regulation of breast cancer cell $PGE_2$ production seems warranted. The apparent disparity between the high breast tumour tissue $PGE_2$ levels previously reported and the inability of most human breast cancer cell lines to synthesise $PGE_2$ remains a paradox. However, this finding is not exceptional since previous studies have also described dramatic differences in $PGE_2$ production between cell lines and primary tumours from specific tissues, including those derived from human lung, colon, prostate and ovarian tumours (Hubbard *et al.*, 1988). Again despite the fact that these tumours are also characterised by elevated $PGE_2$ levels (Karim and Rao, 1976), the majority of these cell lines with the exception of certain non-small-cell carcinomas of the lung, produced little or no detectable $PGE_2$ (Hubbard *et al.*, 1988; 1989). Nonetheless, it is possible that, despite the high exogenous arachidonate concentrations used in the present study, sequestration of substrate via transacylation of phospholipid esters may predominate in some breast cancer cells and prevent metabolism via cyclo-oxygenase. Alternatively, rapid metabolic inactivation of any $PGE_2$ formed by the cancer cells may also limit its detection. Whether the duration of passage affects the capacity of some cell lines to synthesise PG is unknown. However, CLF-90-1 cells, from an early-passage hormone-dependent breast cancer cell line recently established in our laboratory also fail to synthesise $PGE_2$. Notwithstanding our present findings, caution must of course be exercised when interpreting and extrapolating any such study to tumour cells *in vivo*.

Interleukin 1$\beta$ also caused large increases in breast fibroblast $PGE_2$ production in the absence and presence of exogenous substrate. Hence in addition to cyclo-oxygenase induction, the cytokine may also increase phospholipase $A_2$ activity as described in other cell types (Lyons-Giordano *et al.*, 1993). In the absence of added arachidonate, the fibroblast $PGE_2$ response to IL-1$\beta$ was greatly enhanced by BK and ET-1, both of which are known to stimulate phospholipase $A_2$ in several cell types. Since cyclo-oxygenase activity in breast fibroblasts appeared to be unaltered by these peptides, this synergism between peptide and cytokine may be partly mediated by the concerted activation and induction of phospholipase $A_2$ and cyclo-oxygenase respec-

**Figure 4** Effect of phorbol ester and cycloheximide on $PGE_2$ metabolism by T-47D breast cancer cells. T-47D cells were incubated for 24 h with or without TPA (10 nM) or cycloheximide (CHX; 1 μM) and the metabolism of [$^3$H]$PGE_2$ (1 μM) was then monitored during the last 4 h period. Incorporation of radiolabel into various product bands was then determined following extraction and TLC (see Materials and methods). (**a**) Shows typical radioactivity profiles from single incubations of control (○), TPA (●) and cycloheximide (▲) treated cells. (**b**) Shows means ± s.d. values ($n = 3$) for the sum of the product bands 15-keto $PGE_2$ and PGEM (■) as well as the $PGE_2$ (□) substrate band. *$P<0.02$. **$P<0.01$ for significant changes in relation to basal values.

tively. Similar synergistic interactions between these inflammatory mediators have been described previously for the regulation of PG synthesis in decidual (Schrey *et al.*, 1992*b*; Schrey and Hare, 1992), gingival (Lerner and Modeer, 1991) and synovial fibroblasts (Bathon *et al.*, 1989). The observed inhibition of IL-1β action in breast fibroblasts by dexamethasone would also be characteristic of and consistent with a role for COX-2 induction by the cytokine in these cells as recently described during IL-1β-stimulated PG production in other cell types (Crofford *et al.*, 1994).

The significance of this stromal cell $PGE_2$ production in response to inflammatory kinins and cytokines with regard to breast tumour biology is at present uncertain. Nonetheless IL-1β-mediated increases in matrix-metalloproteinase activity in the tumour microenvironment may be subject to negative regulation by $PGE_2$ (Case *et al.*, 1990; Mackay *et al.*, 1992). We have previously proposed a mediatory autocrine role for $PGE_2$ in the inhibition of breast fibroblast growth by BK (Patel and Schrey, 1992). Interestingly, in this regard BK has been reported to inhibit the *in vivo* growth of spontaneous mammary tumours and sarcomas in mice (Mashiba and Matsunaga, 1985) and SV-40-induced fibrosarcomas in hamsters (Koppelmann *et al.*, 1978). Stromal $PGE_2$ may also exert an inhibitory paracrine influence on breast cancer cell growth as evidenced by its anti-proliferative action on MCF-7 cells (Fentiman *et al.*, 1984).

Notwithstanding an equivocal role for inflammatory mediators such as IL-1β and BK in breast cancer, tumour stroma generation exhibits many parallels with the processes involved in inflammation, connective tissue remodelling and wound repair, including infiltration of cytokine-producing host immune cells (Whalen, 1990). Furthermore, a number of studies indicate a local activation of the kallikrien–kinin cascade in tumours from several different cancers (Maeda *et al.*, 1988; Karlsrud *et al.*, 1991; Matsumura *et al.*, 1991).

A role for ET-1 as a paracrine mitogen for breast stromal tissue has been recently implicated (Baley *et al.*, 1990; Schrey *et al.*, 1992*a*). This action may partially account for the extensive desmoplasia seen in breast tumours (Yamashita *et al.*, 1991, 1992). In the present study we have presented evidence for a novel potential paracrine cross-talk between epithelial and stromal breast tumour components involving ET-1 and $PGE_2$. Thus, ET-1 stimulates $PGE_2$ production by breast fibroblasts and $PGE_2$ stimulates ET-1 production by breast cancer cells. Whether such a positive feedback loop might contribute to the elevated levels of $PGE_2$ and ET-1 seen in breast tumours and to the control of tumour stroma generation remains speculative.

In an attempt to investigate a potential paracrine control of breast stromal cell PG production, co-cultures of T-47D (or MCF-7) cells and breast fibroblasts were employed. Under these co-culture conditions both basal and IL-1β-stimulated stromal cell $PGE_2$ production appeared to be inhibited. Experiments indicating the lack of an inhibitory effect of T47-D cell-conditioned media on fibroblast $PGE_2$ and a reduced recovery of exogenous $PGE_2$ from T47-D cell cultures are consistent with metabolic inactivation of $PGE_2$ by the breast cancer cells. This interpretation was further supported by the metabolic capacity of T47-D cells to readily convert $PGE_2$ to 15-keto-$PGE_2$. 15-Hydroxy-PG dehydrogenase, which catalyses this conversion, is considered to be the key enzyme controlling the biological inactivation of prostaglandins. Although this enzyme appears to be ubiquitous in mammalian tissues, the specific cell types responsible for prostaglandin catabolism are uncertain. Clearly, the regulation of this enzyme may also play an important role in determining breast tumour tissue $PGE_2$ levels. The production of 15-keto-$PGE_2$ in T47-D cells was stimulated by the phorbol ester TPA and inhibited by cycloheximide treatment. In accordance with a recent study using the leukaemia HL-60 cell line to investigate 15-hydroxy PG dehydrogenase regulation (Xun *et al.*, 1991) our observations would be consistent with an induction of the enzyme in T47-D cells by TPA. Similarily, previous studies have also indicated a loss of enzyme activity following cycloheximide treatment consistent with a rapid turnover of the dehydrogenase in lung, kidney (Blackwell *et al.*, 1975) and HL-60 cells (Xun *et al.*, 1991). Since maintenance of dehydrogenase activity appears to require continued *de novo* enzyme synthesis (Xun *et al.*, 1991) a susceptibility to hormonal control might be expected. This is indeed the case with regard to the steroidal regulation of this PG dehydrogenase in the uterine decidua, where progestins appear to induce the enzyme (Alam *et al.*, 1976) and anti-progestins are particularly effective in suppressing enzyme activity and elevating *in situ* PG concentrations

(Cheng *et al.*, 1993). The potential for similar hormonal regulation of this enzyme in breast tissue requires investigation. Indeed, $PGE_2$ levels in DMBA-induced rat mammary tumours increase following ovariectomy (Foecking *et al.*, 1982) and prostaglandins have also been implicated in mastalgia associated with premenstrual syndrome (Budoff, 1986).

Despite the association of elevated $PGE_2$ with a malignant metastatic phenotype, the value of breast tumour $PGE_2$ levels as a prognostic indicator remains uncertain. Although initial studies suggested a poor prognosis in patients with high PG levels (Bennett *et al.*, 1977) others have shown either no relationship to established prognostic indices (Wilson *et al.*, 1980; Watson *et al.*, 1987) or a correlation with favourable prognostic factors (Karmali *et al.*, 1983; Watson and Chuah, 1985; Fulton *et al.*, 1982). These disparate findings could be attributed to various factors including: differences in the techniques employed; substrate availability; and tissue handling as well as the labile nature of both tissue $PGE_2$ levels *per se* and the rapid turnover of the enzymes regulating $PGE_2$ production and metabolism. Interestingly, a recent study measuring immunoreactive membrane-associated phospholipase $A_2$ in breast tissue reported high expression in malignant *vs* benign or normal tissue (Yamashita *et al.*, 1993). Whether this overexpression of phospholipase $A_2$ in malignant breast tissue is instrumental in causing elevated $PGE_2$ levels in these tumours remains to be established.

In summary, this study identifies the breast stromal fibroblast as a potential source for $PGE_2$ production in breast tumours. Putative paracrine factors which may regulate this arachidonate metabolism include cytokines and peptides such as IL-1$\beta$, kinins and endothelin. We are currently engaged on a comparative study to determine whether fibroblasts derived from normal or malignant breast tissue exhibit any differential capacity for $PGE_2$ synthesis. Since the capacity for $PGE_2$ production in breast cancer cells appears to be restricted to oestrogen receptor-negative cell lines with an invasive phenotype, whether the cancer cells *per se* contribute towards prostaglandin synthesis may depend on the specific histological class of tumour. Finally, metabolic inactivation by the cancer cells may also play an important regulatory role in the control of breast tumour $PGE_2$ levels.

### Abbreviations

HBF, human breast fibroblast; PG, prostaglandin; PGEM, prostaglandin E metabolite; IL-1$\beta$, interleukin 1$\beta$; BK, bradykinin; ET-1, endothelin-1; TPA, 12-*O*-tetradecanoylphorbol-13-acetate; PKC, protein kinase C; COX-2, cyclo-oxygenase 2; TLC, thin layer chromatography; EMEM, Eagle's minimal essential medium; FCS, fetal calf serum.

### Acknowledgements

This work was supported in part by grants from Cancer Research Campaign UK and Joint Standing Research Committee, St Mary's Hospital, London.

### References

ALAM AA, RUSSELL PT, TABOR MW AND MOULTON BC. (1976). Progesterone and oestrogen control of uterine prostaglandin dehydrogenase activity during deciduomal growth. *Endocrinology*, **98**, 859-863.

BALAKRISHNAN A, CRAMER S, BANDYOPADHYAY GK, IMAGAWA W, YANG J, ELIAS J, BEATTIE CW, DAS GUPTA TK AND NANDI S. (1989). Differential proliferative response to linoleate in cultures of epithelial cells from normal human breast and fibroadenomas. *Cancer Res.*, **49**, 857-862.

BALEY PA, RESINK TJ, EPPENBERGER U AND HAHN AW. (1990). Endothelin messenger RNA and receptors are differentially expressed in cultured human breast epithelial and stromal cells. *J. Clin. Invest.*, **875**, 1320-1323.

BANDYOPADHYAY GK, IMAGAWA W, WALLACE D AND NANDI S. (1987). Linoleate metabolites enhance the *in vitro* proliferative response of mouse mammary epithelial cells to epidermal growth factor. *J. Biol. Chem.*, **262**, 2750-2756.

BATHON JM, PROUD P, KRACKOW K AND WIGLEY FM. (1989). Pre-incubation of human synovial cells with interleukin-1 modulates prostaglandin $E_2$ release in response to bradykinin. *J. Immunol.*, **143**, 579-586.

BENNETT A. (1979). Prostaglandins and cancer. In *Practical Applications of Prostaglandins and their Inhibitors*, Karim SMM (ed.) pp. 149-162. Raven Press: New York.

BENNETT A, CHARLIER EM, McDONALD AM, SIMPSON, STAMFORD IF AND ZEBRO T. (1977). Prostaglandins and breast cancer. *Lancet*, **2**, 624-626.

BLACKWELL GJ, FLOWER GJ AND VANE JR. (1975). Rapid reduction of prostaglandin 15-hydroxy dehydrogenase activity in rat tissues after treatment with protein synthesis inhibitors. *Br. J. Pharmacol.*, **55**, 233-238.

BUDOFF PW. (1986). Premenstrual syndrome. In *Prostaglandins and their Inhibitors in Clinical Obstetrics and Gynaecology*, Bygdeman M, Berger GS and Keith LG (eds) pp. 367-390. MTP Press: Lancaster.

BURCH RM AND CONNOR JR. (1992). Elevated cAMP is required for stimulation of eicosanoid synthesis by interleukin 1 and bradykinin in balb/c3T3 fibroblasts. *J. Cell. Physiol.*, **151**, 512-518.

CASE JP, LAFYATIS R, KUMKUMIAN GK, REMMERS EF AND WILDER RL. (1990). IL-1 regulation of transin/stromelysin transcription in rheumatoid synovial fibroblasts appears to involve two antagonistic transduction pathways, an inhibitory, prostaglandin-dependent pathway mediated by cAMP and a stimulatory protein kinase C-dependent pathway. *J. Immunol.*, **145**, 3755-3761.

CHENG L, KELLY RW, THONG KJ, HUME R AND BAIRD DT. (1993). Effect of mifepristone (RU486) on prostaglandin dehydrogenase in decidual and chorinoic tissue in early pregnancy. *Hum. Reprod.*, **8**, 705-709.

CROFFORD LJ, WILDER RL, RISTIMAKI AP, SANO H, REMMERS EF, EPPS HR AND HLA T. (1994). Cyclooxygenase-1 and -2 expression in rheumatoid synovial tissues. Effects of interleukin-1 and phorbol ester and corticosteroids. *J. Clin. Invest.*, **93**, 1095-1101.

FENTIMAN IS, DUHIG T, GRIFFITHS AB AND TAYLOR-PAPADIMITRIOU J. (1984). Cyclic AMP inhibits the growth of human breast cancer cells in defined medium. *Mol. Biol. Med.*, **2**, 81-88.

FERNIG DG, SMITH JA AND RUDLAND PS. (1990). Relationship of growth factors and differentiation in normal and neoplastic development of the mammary gland. In *Regulatory Mechanisms in Breast Cancer*, Lippman ME and Dickson RB (eds) pp. 47-78. Kluwer Academic: Boston.

FOECKING MK, KIDDEY WE, ABOU-ISSA H, MATTHEWS RH AND MINTON JP. (1982). Hormone dependence of 7, 12-dimethylbenz(a)anthracene-induced mammary tumour growth; correlation with prostaglandin $E_2$ content. *J. Natl Cancer Inst.*, **69**, 443-446.

FULTON AM AND HEPPNER GH. (1985). Relationship of prostaglandin E and natural killer sensitivity to metastatic potential in murine mammary adenocarcinoma. *Cancer Res.*, **45**, 4779-4784.

FULTON A, ROI L, HOWARD L, RUSSO J, BROOKS S AND BRENNAN MJ. (1982). Tumour-associated prostaglandins in patients with primary breast cancer. Relationship to clinical parameters. *Breast Cancer Res. Treat.*, **2**, 331-337.

FULTON AM, ZHANG S-Z AND CHONG YC. (1991). Role of the prostaglandin $E_2$ receptor in mammary tumour metastasis. *Cancer Res.*, **51**, 2047-2050.

GORMAN RR, BIENKOWSKI MJ AND LIN AH. (1991). Elevated prostaglandin H synthase gene expression in ras-transformed cells. *Adv. Prostaglandin Thromboxane Leukotriene. Res.*, **21A**, 73-76.

HUBBARD WC, ALLEY MC, McLEMORE TL AND BOYD MR. (1988). Profiles of prostaglandin biosynthesis in sixteen established cell lines derived from human lung, colon, prostate and ovarian tumours. *Cancer Res.*, **48**, 4770-4775.

HUBBARD WC, ALLEY MC, GRAY GN, GREEN KC, McLEMORE TL AND BOYD MR. (1989). Evidence for prostanoid biosynthesis as a biochemical feature of certain subclasses of non-small cell carcinoma of the lung as determined in established cell lines derived from human lung tumours. *Cancer Res.*, **49**, 826-832.

KARIM SMM AND RAO B. (1976). Prostaglandins and tumours. In *Advances in Prostaglandin Research*, Vol. 2, SMM Karim (ed.), pp. 302–325. MTP Press: Boston.

KARLSRUD TS, BUO L, AASEN AO AND JOHANSEN HT. (1991). Characterization of kininogens in human malignant ascites. *Thromb. Res.*, **63**, 641–650.

KARMALI RA, WELT S, THALER HT AND LEFEVRE F. (1983). Prostaglandins in breast cancer. Relationship to disease stage and hormone status. *Br. J. Cancer*, **48**, 689–696.

KELLY RW, DEAM S, CAMERON MJ AND SEAMARK RF. (1986). Measurement by radioimmunoassay of prostaglandins as their methyl oximes. *Prostaglandins Leukotrienes Essential Fatty Acids*. **24**, 1–14.

KOPPELMANN LE, MOORE TC AND PORTER D. (1978). Increased plasma kallikrein activity and tumor growth suppression associated with intralesional bradykinin injection in hamsters. *J. Pathol.*, **126**, 1–10.

KOZMA S, BOGGARD M, BUSER K, SAUER K, BOS J, GRONER B AND HYNES N. (1987). The human c-Kirsten ras gene is activated by a novel mutation in codon 13 in the breast carcinoma cell line MDA-MD-231. *Nucleic Acid Res.*, **15**, 5963–5970.

KRAUS M, YUASA Y AND AARONSON SA. (1984). A position 12-activated H-ras oncogene in all Hs578T mammary carcinoma cells but not normal mammary cells of the same patient. *Proc. Natl Acad. Sci. USA*, **81**, 5384–5388.

LERNER UH AND MODEER T. (1991). Bradykinin $B_1$ and $B_2$ receptor agonists synergistically potentiate interleukin-1 induced prostaglandin biosynthesis in human gingival fibroblasts. *Inflammation*, **15**, 427–436.

LOWRY OH, ROSEBROUGH NJ, FARR AL AND RANDALL RJ. (1951). Protein measurement with the Folin phenol reagent. *J. Biol. Chem.*, **193**, 265–275.

LYONS-GIORDANO B, PRATTA MA, GALBRAITH W, DAVIS GL AND ARNER EC. (1993). Interleukin-1 differentially modulates chondrocyte expression of cyclooxygenase-2 and phospholipase $A_2$. *Exp. Cell. Res.*, **206**, 58–62.

MACKAY AR, BALLIN M, PELINA MD, FARINA AR, NASON AM, HARTZLAE JL AND THORGEIRSSON UP. (1992). Effect of phorbol ester and cytokines on matrix metalloproteinase and tissue inhibitor of metalloproteinase expression in tumor and normal cell lines. *Invasion Metastasis*, **12**, 168–184.

MAEDA H, MATSUMURA Y AND KATO H. (1988). Purification and identification of [Hyp³]-bradykinin in ascitic fluid from a patient with gastric cancer. *J. Biol. Chem.*, **63**, 16051–16056.

MASHIBA H AND MATSUNAGA K. (1985). Tumor-inhibitory effect of intralesional injection of bradykinin and immunostimulants in mice. *Cancer Lett.*, **29**, 177–182.

MATSUMURA Y, MARUO K, KIMURA M, YAMAMOTO T, KONNO T AND MAEDA H. (1991). Kinin-generating cascade in advanced cancer patients: an in vitro study. *Jpn. J. Cancer Res.*, **82**, 732–741.

MITCHELL BF, ROGERS K AND WONG S. (1993). The dynamics of prostaglandin metabolism in human fetal membranes and decidua around the time of parturition. *J. Clin. Endocrinol. Metab.*, **77**, 759–764.

PACIOTTI GF AND TAMARKIN L. (1988). Interleukin-1 directly regulates hormone-dependent human breast cancer cell proliferation *in vitro*. *Mol. Endocrinol.*, **2**, 459–464.

PATEL KV AND SCHREY MP. (1990). Activation of inositol phospholipid signalling and $Ca^{++}$ efflux in human breast cancer cells by bombesin. *Cancer Res.*, **52**, 334–340.

PATEL KV AND SCHREY MP. (1992). Inhibition of DNA synthesis and growth by bradykinin in human breast stromal cells. Evidence for independent roles for $B_1$ and $B_2$ receptors in the respective control of cell growth and phospholipid hydrolysis. *Cancer Res.*, **52**, 334–340.

PATEL KV AND SCHREY MP. (1995). Human breast cancer cells contain a phosphoramidon-sensitive metalloproteinase which can process exogenous big endothelin-1 to endothelin-1: a proposed mitogen for human breast fibroblasts. *Br. J. Cancer*, **71**, 442–447.

RAZ A, WYCHE A, FU J, SEIBERT K AND NEEDLEMAN P. (1990). Regulation of prostanoid synthesis in human fibroblasts and human blood monocytes by interleukin-1, endotoxin and glucocorticoids. *Adv. Prostaglandin Thromboxane and Leukotriene. Res.*, **20**, 22–27.

ROLLAND PH, MARTIN PM, JACQUEMIER J, ROLLAND A AND TOGA M. (1980). Prostaglandins in human breast cancer: Evidence suggesting that an elevated prostaglandin production is a marker of high metastatic potential for neoplastic cells. *J. Natl Cancer Inst.*, **64**, 1061–1070.

SCHREY MP AND HARE A. (1992). Endothelin-1 stimulates phospholipid hydrolysis and prostaglandin $F_{2\alpha}$ production in primary human decidua cell cultures. *Prostaglandins Leukotrienes Essential Fatty Acids*. **47**, 321–325.

SCHREY MP, PATEL KV AND TEZAPSIDIS N. (1992*a*). Bombesin and glucocorticoids stimulate human breast cancer cells to produce endothelin, a paracrine mitogen for breast stromal cells. *Cancer Res.*, **52**, 1786–1790.

SCHREY MP, HOLT JR, CORNFORD PA, MONAGHAN H AND AL-UBAIDA F. (1992*b*). Human decidua is a target for bradykinin and kallikrein: phosphoinositide hydrolysis accompanies arachidonic acid release in uterine decidua cells *in vitro*. *J. Clin. Endocrinol. Metab.*, **74**, 426–435.

TAN WC, PRIVETT OS AND GOLDYNE ME. (1974). Studies of prostaglandins in rat mammary tumours induced by 7,12-dimethylbenz(a)anthracene. *Cancer Res.*, **34**, 3229–3231.

THOMPSON EW, PAIK S, BRUNNER S, SOMMERS CL, ZUGMAIER G, CLARKE R, SHIMA TB, TORRI J, DONAHUE S, LIPPMAN ME, MARTIN GR AND DICKSON RB. (1992). Association of increased basement membrane invasiveness with absence of estrogen receptor and expression of vimentin in human breast cancer cell lines. *J. Cell. Physiol.*, **150**, 534–544.

WHALEN GF. (1990). Solid tumours and wounds: transformed cells misunderstood as injured tissue? *Lancet*, **336**, 1489–92.

WATSON DMA, KELLY RW AND MILLER WR. (1987). Prostaglandins and prognosis in human breast cancer. *Br. J. Cancer*, **56**, 367–370.

WATSON J AND CHUAH SY. (1985). Prostaglandins, steroids and human mammary cancer. *Eur. J. Cancer Clin. Oncol.*, **21**, 1051–1055.

WATSON J AND CHUAH SY. (1992). Technique for the primary culture of human breast cancer cells and measurement of their prostaglandin secretion. *Clinical Science*, **83**, 347–352.

WILSON AI, BAUM N, BENNETT A, GRIFFITHS K, NICHOLSON RI AND STAMFORD IF. (1980). Lymph node status, prostaglandin and oestrogen receptors are independent variables in human primary breast cancer. *Clin. Oncol.*, **6**, 379–387.

XUN C-Q, TIAN Z-G AND TAI H-H. (1991). Stimulation of synthesis de novo of NAD+-dependent 15-hydroxyprostaglandin dehydrogenase in human promyelocytic leukaemia (HL-60) cells by phorbol ester. *Biochem. J.*, **279**, 553–558.

YAMASHITA J-I, OGAWA M, INADA K, YAMASHITA S-I, MATSUO S AND TAKANO S. (1991). A large amount of endothelin-1 is present in human breast cancer tissue. *Res. Commun. Chem. Pathol. Pharmacol.*, **74**, 363–370.

YAMASHITA J-I, OGAWA M, EGAMI H, MATSUO S, KIYOHARA H, INADA K, YAMASHOTA S-I AND FUJITA S. (1992). Abundant expression of immunoreactive endothelin 1 in mammary phyllodes tumors: Possible role of endothelin 1 in the growth of stromal cells in phyllodes tumor. *Cancer Res.*, **52**, 4046–4049.

YAMASHITA S-I, YAMASHITA J-I, SAKAMOTO K, INADA K, NAKASIMA Y, MURATA K, SAISHOJI T, NORURA K AND OGAWA M. (1993). Increased expression of membrane-associated phospholipase $A_2$ shows malignant potential of human breast cancer cells. *Cancer*, **71**, 3058–3064.